# Irregular Pulsation of Intracranial Aneurysm Detected by Four-Dimensional CT Angiography and Associated With Small Aneurysm Rupture: A Single-Center Prospective Analysis

**DOI:** 10.3389/fneur.2022.809286

**Published:** 2022-02-24

**Authors:** Jiafeng Zhou, Qinhua Guo, Yongchun Chen, Boli Lin, Shenghao Ding, Huilin Zhao, Yaohua Pan, Jieqing Wan, Bing Zhao

**Affiliations:** ^1^Department of Neurosurgery, Renji Hospital, Shanghai Jiaotong University School of Medicine, Shanghai, China; ^2^Department of Radiology, The First Affiliated Hospital of Wenzhou Medical University, Wenzhou, China; ^3^Department of Radiology, Renji Hospital, Shanghai Jiaotong University School of Medicine, Shanghai, China

**Keywords:** intracranial aneurysm, small aneurysms, rupture, risk factor, 4D-CT angiography

## Abstract

**Objectives:**

Predicting the risk of rupture of small intracranial aneurysms remains challenging. The irregular pulsation of aneurysms detected by four-dimensional CT angiography (4D-CTA) could be an imaging marker of aneurysm vulnerability. We aimed to investigate the association of irregular pulsation with small aneurysm rupture.

**Materials and Methods:**

This was a prospective study on intracranial aneurysms detected by 4D-CTA from October 2017 to January 2020. A total of 242 consecutive patients with 316 aneurysms were enrolled. Irregular pulsation was defined as a temporary focal protuberance on more than 3 consecutive frames of the 20 phases in the RR interval. Small aneurysms were defined as those <7 mm. Univariate and multivariate analyses were performed to determine the independent predictors of small aneurysm rupture.

**Results:**

A total of 169 patients with 217 small intracranial aneurysms were included. Fourteen (6.5%) of the aneurysms had ruptured, and 77 (35.5%) had irregular pulsation. There were no significant differences in age, sex, hypertension, smoking, diabetes, drinking, or hyperlipidemia between the ruptured and unruptured aneurysm groups. The univariate analysis showed that smaller vessel size (*p* = 0.008), larger size ratio (*p* = 0.003), larger aspect ratio (*p* = 0.006), larger flow angle (*p* = 0.001), large vessel angle (*p* = 0.004), middle cerebral artery aneurysms (*p* = 0.046), anterior cerebral artery/posterior communicating artery/posterior circulation aneurysm (*p* = 0.006), irregular aneurysm (*p* = 0.001), and t presence of irregular pulsation (*p* = 0.001) were associated with small aneurysm rupture. The multivariate analysis showed that the presence of irregular pulsation (*p* = 0.003), anterior cerebral artery/posterior communicating artery/posterior circulation aneurysms (*p* = 0.014), and larger flow angle (*p* = 0.006) was independently associated with aneurysm rupture. Multivariate analysis of predictors of the irregular pulsation of small aneurysms showed that the aneurysm rupture (*p* = 0.022), irregular aneurysm (*p* < 0.001), and large size ratio (*p* = 0.005) were independently associated with the presence of irregular pulsation.

**Conclusions:**

The ruptured small aneurysms more often had irregular pulsation. The irregular pulsation was independently associated with aneurysm rupture and may help evaluate the risk of rupture of small intracranial aneurysms.

## Introduction

Unruptured intracranial aneurysms (UIAs) are increasingly being detected as non-invasive imaging modalities are commonly used. Although a wealth of data is available for the natural history of UIAs, this history remains poorly understood, especially that of small (<7 mm) aneurysms (1–3). Majority of patients with small UIAs have low risk of rupture (1, 3, 4). However, small aneurysms have a high proportion in patients with subarachnoid hemorrhage (SAH) (5, 6). Therefore, there is a controversy regarding which small UIAs can be left untreated, or which aneurysms are needed to be treated (4, 7). Conventional morphological parameters, such as aneurysm irregularity, aneurysm location, and size ratio, are associated with aneurysm rupture (4, 6); however, these parameters are confounding and controversial (8). Four-dimensional CT angiography (4D-CTA) can acquire time-resolved three-dimensional reconstructions to get geometric and hemodynamic information on and quantification of aneurysm volume changes during cardiac cycle (9–11). Recent studies have reported that irregular pulsation observed by 4D-CTA could be a novel imaging marker of aneurysm vulnerability (12–14).

In this study, we performed a prospective study on small intracranial aneurysms detected by 4D-CTA. We investigated the association of irregular pulsation of aneurysms with ruptured aneurysms and investigated the relationships between irregular pulsation and conventional risk factors.

## Materials and Methods

We conducted this study in accordance with the Declaration of Helsinki. It was approved by the Institutional Review Board of Renji Hospital, School of Medicine, Shanghai Jiao Tong University. Written informed consent was obtained.

### Study Design and Patients

This was a prospective study on 242 consecutive patients with 316 aneurysms who underwent 4D-CTA and were enrolled from October 2017 to January 2020. 4D-CTA is a routine examination for patients with intracranial aneurysms in our hospital. Demographic and clinical information, including sex, age, hypertension, smoking history, drinking, hyperlipidemia, diabetes, clinical conditions, and radiological findings were prospectively collected. Inclusion criteria are the following: (1) patients between 18 and 85 years of age; (2) all intracranial aneurysms were confirmed by digital subtraction angiography (DSA); (3) patients or their families signed informed consent forms. Exclusion criteria are the following: (1) women who are pregnant or breastfeeding; (2) patients combined with other refractory diseases such as tumors and hematologic diseases; (3) patients with other severe systemic diseases such as acute myocardial infarction, renal dysfunction, and malignant tumors. In this study, small aneurysms were defined as those <7 mm in diameter. A ruptured aneurysm was defined as an aneurysm that was responsible for subarachnoid hemorrhage. If there were multiple aneurysms, a ruptured aneurysm was considered according to the clinical condition and initial CT scan. Cavernous carotid aneurysms, giant aneurysms, recurrent aneurysms, aneurysms whose images cannot be reconstructed because of poor quality, and aneurysms ≥7 mm in diameter were further excluded for the final analysis.

### 4D-CTA Protocol and Irregular Pulsation Definition

**Figure 1:**
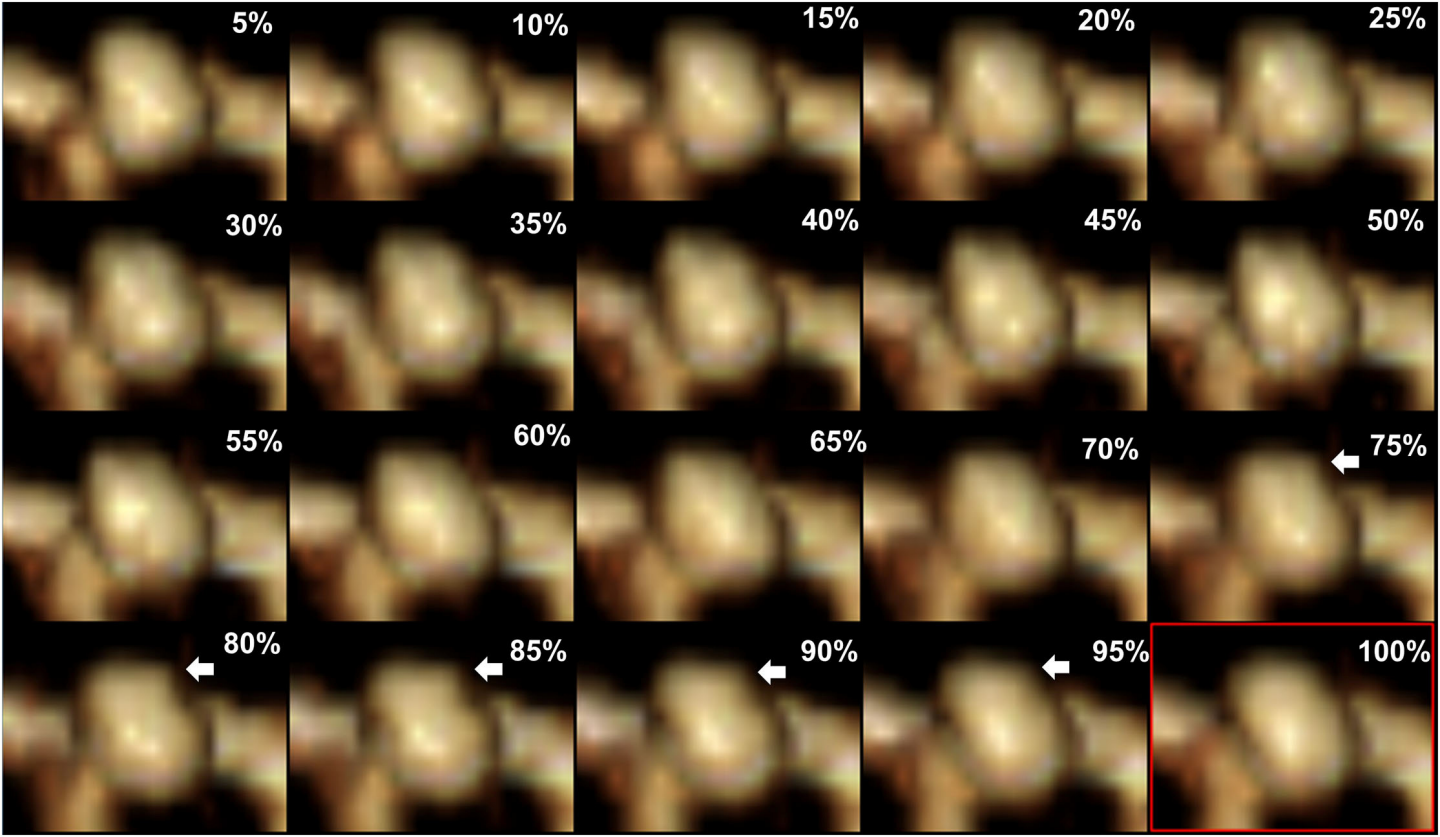
Aneurysm irregular pulsation detected by four-dimensional CT angiography reconstruction. A focal protuberance occurs at the dome of the aneurysm in the 15–19th frames corresponding to the 20 phases in the R-R interval in a single cardiac cycle (white arrow). The protuberance disappears in the 20th frame (red rectangle).

4D-CTA imaging was performed using a 320-detector row CT scanner (Aquilion One; Toshiba Medical Systems Corporation, Otawara, Japan) with an electrocardiographic (ECG)-gated scan mode during a single cardiac cycle. The imaging protocol was published previously elsewhere (14). Slice thickness 0.5 mm, 120 kV/230 mA, 275 ms gantry rotation time, 16-cm long field-of-view, and resolution of 0.31 × 0.31 × 0.5 mm were used as scanning parameters. Injection of a 60-ml iodinated contrast medium at 3–4 ml/s (iomeprol 400 mg/ml; Iomeron; Bracco, Milan, Italy) was performed as 4D-CTA acquisition. The RR interval was divided into 20 phases at 5% intervals for ECG-gated reconstruction, and irregular pulsation was defined as a temporary focal protuberance on more than 3 consecutive frames of the 20 phases in the R-R interval (14) (Figure 1). Irregular pulsation was prospectively evaluated by two neuroradiologists who were blinded to the rupture status of aneurysms. In case of disagreement between the two reviewers, a third experienced neuroradiologist was consulted for final interpretation.

### Aneurysm Morphology and Measurement

**Figure 2:**
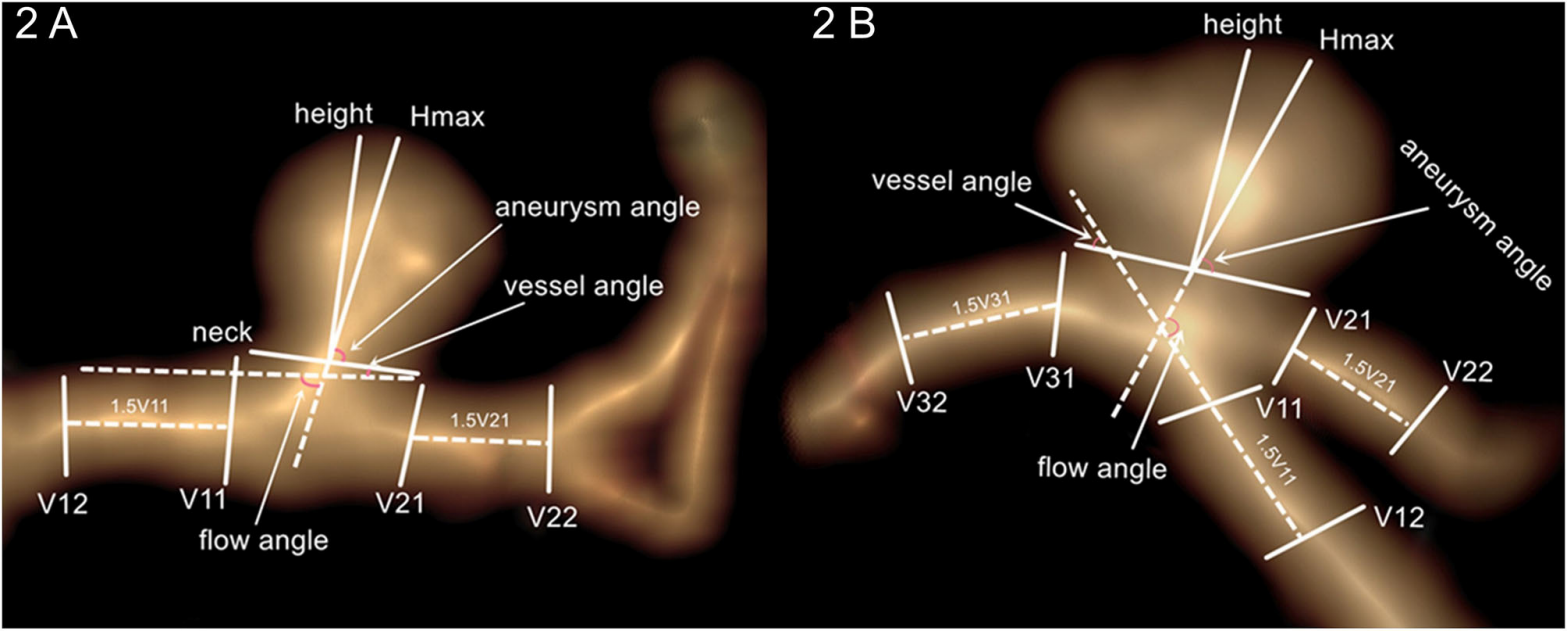
Aneurysm morphology measurement. **(A)** Intracranial side wall aneurysm measurement. **(B)** Intracranial bifurcation aneurysm measurement.

A workstation (Version 4.4; GE Medical Systems) was used to reconstruct 3D images and measure aneurysm morphology. Aneurysm morphological parameters, namely, aneurysm height, perpendicular height, aneurysm size, neck size, vessel size, aspect ratio, size ratio, aneurysm angle, flow angle, and vessel angle were described in detail in previous studies (15, 16). The aneurysm size was the largest cross-sectional diameter of the aneurysm dome. Aneurysm height was the greatest distance from the dome to the center of aneurysm neck. Vessel size was the mean size of vessels surrounding an aneurysm. Aspect ratio was the ratio of perpendicular height to neck size. Size ratio was the ratio of aneurysm height to vessel size. Vessel angle was the angle between the vector of blood flow and the plane of aneurysm neck. Flow angle was the angle between the blood flow of the parent artery and aneurysm height line. These aneurysm parameters are measured, and the descriptions are shown in Figure 2.

### Statistical Analysis

A statistical analysis was performed using IBM SPSS V.20.0 (IBM SPSS, Armonk, NY, United States). A *p*-value < 0.05 was regarded as statistically significant. Categorical variables were given as frequencies (percentages), and continuous variables were given as means ± standard deviations. Categorical variables were analyzed by chi-squared test or Fisher's exact test. Continuous variables were analyzed by independent-sample *t*-test or Mann–Whitney *U*-test. Univariate and multivariate logistic regression analyses were performed to investigate risk factors for small aneurysm rupture and determine relationships between irregular pulsation and conventional risk factors. Variables with a *p*-value < 0.05 were entered into the multivariable analysis using the backward LR method, 95% confidence intervals (CIs) and odds ratios (ORs) were calculated. Area under the curve (AUC) was calculated to test the predictive ability of the model.

## Results

### Baseline Characteristics

**Figure 3:**
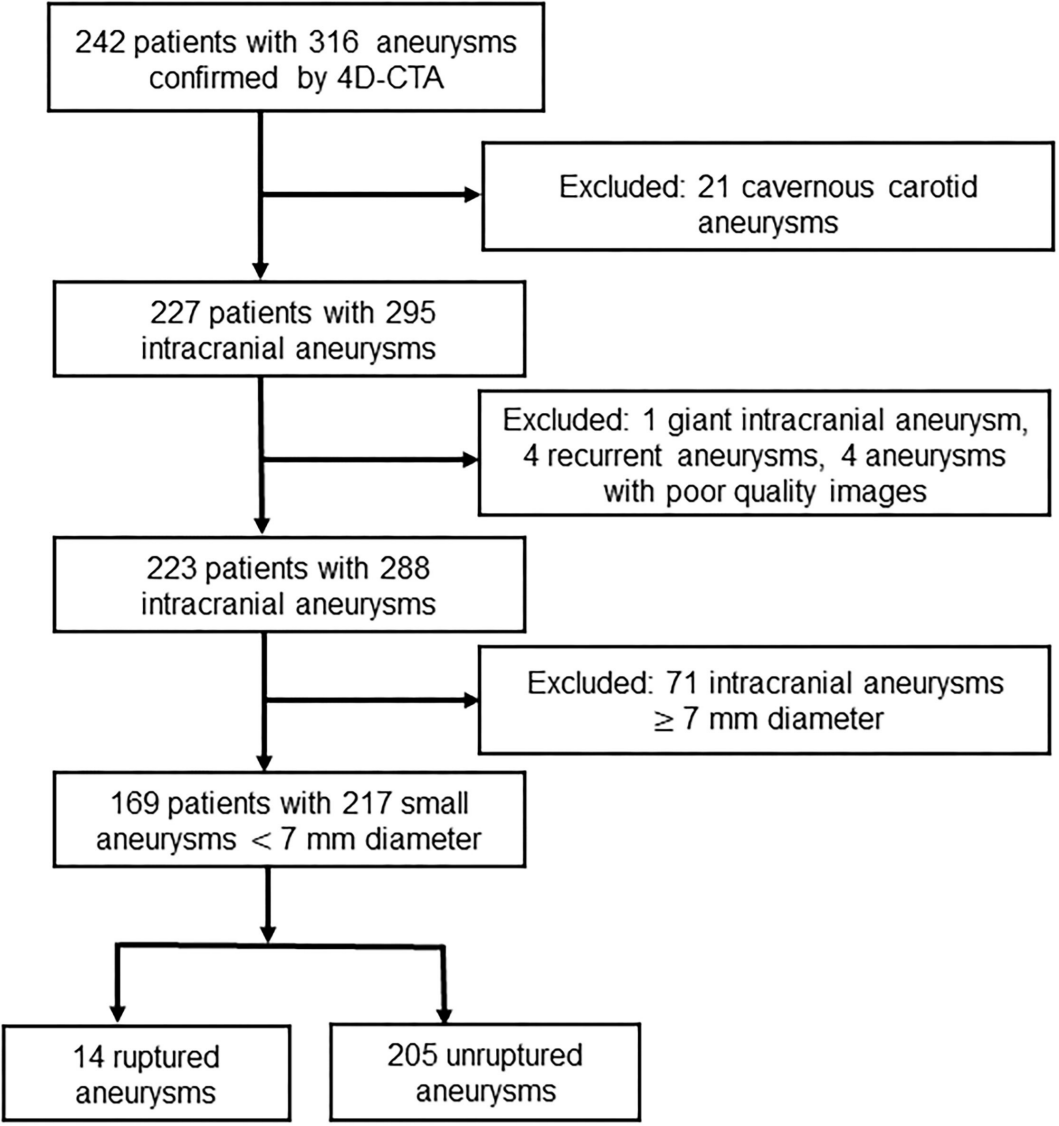
Study flow diagram.

A total of 169 patients with 217 small intracranial aneurysms were included in this study (Figure 3). Mean age was 59.8 ± 10.9 years (27–85 years). Fourteen of the 217 (6.5%) intracranial aneurysms had ruptured, and 77 (35.5%) had irregular pulsation detected by 4D-CTA. Patient demographics of the ruptured and unruptured aneurysm groups are presented in Table 1. There were no significant differences in age, sex, hypertension, smoking, diabetes, drinking, or hyperlipidemia between the two groups. Aneurysm characteristics of the ruptured and unruptured aneurysm groups are presented in Table 2. Ruptured aneurysms more often had larger size ratio (*p* = 0.001), larger aspect ratio (*p* = 0.004), irregular shape (*p* = 0.001), and irregular pulsation (*p* < 0.001). Patient demographic and aneurysm characteristics between aneurysms with irregular pulsation and those without irregular pulsation are presented in Table 3. Irregular pulsation more often occurred in larger aneurysms (*p* < 0.001), aneurysms with larger size ratios (*p* < 0.001) and larger aspect ratios (*p* < 0.001), non-carotid artery aneurysm (*p* = 0.007), irregular aneurysms (*p* < 0.001), and ruptured aneurysms (*p* < 0.001).

**Table 1:** Patient characteristics between the unruptured and ruptured aneurysm groups in patients with small intracranial aneurysms.

**Variables**	***n* = 169**	**Unruptured (*n* = 155)**	**Ruptured (*n* = 14)**	***p-*value**
Age	169	60.1 ± 10.9	58.7 ± 14.6	0.652
Sex (female/male)	169	53/102	6/8	0.515
Hypertension	83	77 (49.7%)	6 (42.9%)	0.625
Smoking	32	28 (18.1%)	4 (28.6%)	0.306
Diabetes	20	19 (12.3%)	1 (7.1%)	1
Drinking	22	19 (12.3%)	3 (21.4%)	0.398
Hyperlipidemia	34	32 (22.1%)	2 (15.4%)	0.736

**Table 2:** Baseline characteristics between the unruptured and ruptured aneurysm groups.

**Variables**	**Small intracranial aneurysms**		***p-*value**
	**Unruptured (*n* = 203)**	**Ruptured (*n* = 14)**	
Aneurysm size (mm)	4.2 ± 1.3	4.5 ± 1.3	0.303
Neck size (mm)	3.3 ± 0.9	2.9 ± 0.9	0.095
Perpendicular height (mm)	2.7 ± 1.2	3.1 ± 0.9	0.225
Aneurysm height (mm)	3.0 ± 1.2	3.6 ± 1.2	0.066
Vessel size (mm)	3.3 ± 0.9	2.6 ± 1.0	0.017
Size ratio	1.0 ± 0.6	1.5 ± 0.6	0.001
Aspect ratio	0.8 ± 0.3	1.1 ± 0.4	0.004
Flow angle	94.7 ± 31.2	126.8 ± 34.9	<0.001
Vessel angle	17.1 ± 22.6	36.9 ± 27.5	0.002
Aneurysm angle	68.4 ± 19.5	67.9 ± 16.4	0.736
Aneurysm location			<0.001
ICA	115 (56.7%)	1 (7.1%)	
MCA	19 (9.4%)	2 (14.3%)	
ACA/PCoA/PC	69 (34.0%)	11 (78.6%)	
Aneurysm shape			0.001
Regular	169 (83.3%)	6 (42.9%)	
Irregular	34 (16.7%)	8 (57.1%)	
Presence of irregular pulsation	65 (32.0%)	12 (85.7%)	<0.001

*ICA, internal carotid artery; MCA, middle cerebral artery; ACA, anterior cerebral arteries (including anterior cerebral artery and anterior communicating artery); PCoA, posterior communicating artery; PC, posterior circulation*.

**Table 3:** Baseline characteristics between aneurysms with irregular pulsation and those without irregular pulsation.

**Variables**	**Irregular pulsation**		***p-*value**
	**Absence (*n* = 140)**	**Presence (*n* = 77)**	
Age	60.2 ± 9.9	59.1 ± 12.7	0.734
Sex (female/male)	98/42	48/29	0.25
Hypertension	62 (44.3%)	41 (53.2%)	0.206
Smoking	20 (14.3%)	19 (24.7%)	0.056
Diabetes	23 (16.4%)	7 (9.1%)	0.134
Drinking	15 (10.4%)	11 (14.1%)	0.448
Hyperlipidemia	23 (17.2%)	17 (23.9%)	0.244
Aneurysm size (mm)	3.9 ± 1.3	4.7 ± 1.1	<0.001
Neck size (mm)	3.2 ± 0.9	3.5 ± 1.0	0.046
Perpendicular height (mm)	2.5 ± 1.2	3.2 ± 1.0	<0.001
Aneurysm height (mm)	2.8 ± 1.2	3.6 ± 1.0	<0.001
Vessel size (mm)	3.4 ± 0.9	3.1 ± 0.9	0.005
Size ratio	0.9 ± 0.5	1.3 ± 0.6	<0.001
Aspect ratio	0.8 ± 0.3	1.0 ± 0.4	<0.001
Flow angle	93.2 ± 31.3	103.3 ± 33.4	0.027
Vessel angle	16.1 ± 22.8	22.5 ± 24.1	0.004
Aneurysm angle	69.6 ± 19.4	66.6 ± 18.5	0.203
Aneurysm location			0.007
ICA	85 (60.7%)	31 (40.3%)	
MCA	9 (6.4%)	12 (15.6%)	
ACA/PCoA/PC	46 (32.9%)	34 (44.2%)	
Aneurysm rupture	2 (1.4%)	12 (15.6%)	<0.001
Aneurysm shape			<0.001
Regular	131 (93.6%)	44 (57.1%)	
Irregular	9 (6.4%)	33 (42.9%)	

*ICA, internal carotid artery; MCA, middle cerebral artery; ACA, anterior cerebral arteries (including anterior cerebral artery and anterior communicating artery); PCoA, posterior communicating artery; PC, posterior circulation*.

### Predictors of Small Aneurysm Rupture

**Figure 4:**
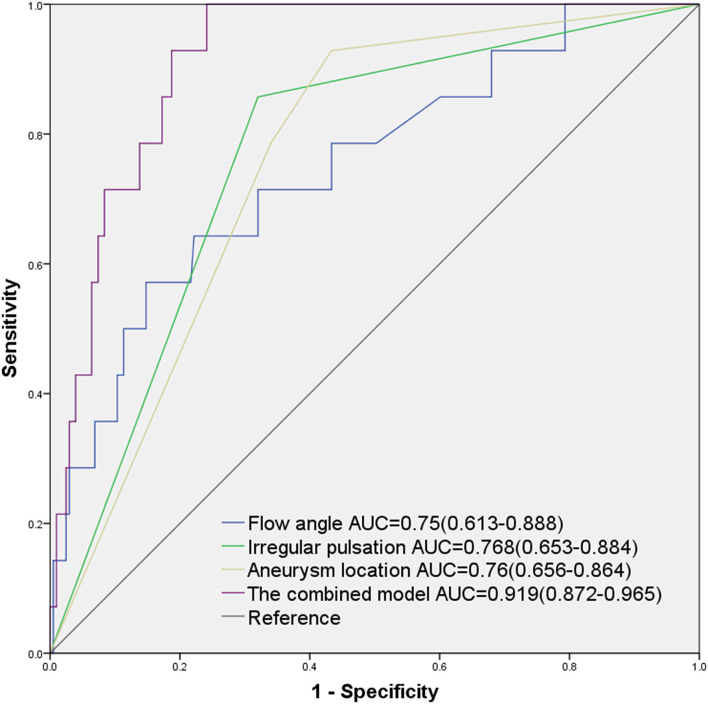
Area under the curves (AUCs) of the predictive value of small aneurysm rupture according to independent predictors.

The univariate and multivariate analyses for predictors of small aneurysm rupture are presented in Table 4. In the univariate analysis, smaller vessel size (*p* = 0.008), larger size ratio (*p* = 0.003), larger aspect ratio (*p* = 0.006), larger flow angle (*p* = 0.001), large vessel angle (*p* = 0.004), middle cerebral artery aneurysm (*p* = 0.046), anterior cerebral artery/posterior communicating artery/posterior circulation aneurysm (*p* = 0.006), irregular aneurysm (*p* = 0.001), and presence of irregular pulsation (*p* = 0.001) were associated with small aneurysm rupture. In the multivariate analysis, larger flow angle (*p* = 0.006), anterior cerebral artery/posterior communicating artery/posterior circulation aneurysms (*p* = 0.014), and presence of irregular pulsation (*p* = 0.003) were independently associated with small aneurysm rupture. The model including flow angle, non-internal carotid artery aneurysm, and irregular pulsation showed good discrimination in predicting small aneurysm rupture with an AUC of 0.919 (0.872–0.965), sensitivity of 100%, and specificity of 75.9% (Figure 4).

**Table 4:** Univariate and multivariate analyses of predictors of small aneurysm rupture.

**Variables**	**Univariable**		**Multivariable**	
	**OR (95% CI)**	***p-*value**	**OR (95% CI)**	***p-*value**
Vessel size	0.45 (0.25–0.81)	0.008		
Size ratio	3.09 (1.46–6.54)	0.003		
Aspect ratio	6.70 (1.71–26.32)	0.006		
Flow angle	1.03 (1.01–1.05)	0.001	1.03 (1.01–1.04)	0.006
Vessel angle	1.03 (1.01–1.04)	0.004		
**Aneurysm location**				
MCA vs. ICA	12.11 (1.05–140.15)	0.046		
ACA/PCoA/PC vs. ICA	18.33 (2.32–145.11)	0.006	14.59 (1.73–122.99)	0.014
Irregular aneurysm	6.63 (2.16–20.33)	0.001		
Presence of irregular pulsation	12.74 (2.77–58.58)	0.001	11.05 (2.21–55.35)	0.003

*MCA, middle cerebral artery; ACA, anterior cerebral arteries (including anterior cerebral artery and anterior communicating artery); PCoA, posterior communicating artery; PC, posterior circulation*.

### Predictors of Irregular Pulsation of Small Aneurysms

The univariate and multivariate analyses for predictors of irregular pulsation of small aneurysms are shown in Table 5. In the univariate analysis, larger aneurysm (*p* < 0.001), wider aneurysm (*p* = 0.048), longer aneurysm (*p* < 0.001), smaller vessel size (*p* = 0.008), larger size ratio (*p* < 0.001), larger aspect ratio (*p* < 0.001), larger flow angle (*p* = 0.029), middle cerebral artery aneurysm (*p* = 0.008), and anterior cerebral artery/posterior communicating artery/posterior circulation aneurysm (*p* = 0.022) were associated with irregular pulsation. In the multivariate analysis, aneurysm rupture (OR 7.05, 95% CI 1.33–37.24, *p* = 0.022), irregular aneurysm (OR 6.44, 95% CI 2.69–15.4, *p* < 0.001), and large size ratio (OR 2.59, 95% CI 1.34–4.99, *p* = 0.005) were independently associated with presence of irregular pulsation. The model including aneurysm rupture, aneurysm irregularity, and size ratio predicted irregular pulsation with an AUC of 0.767 (0.701–0.833), sensitivity of 58.4%, and specificity of 85%.

**Table 5:** Univariate and multivariate analyses of predictors of irregular pulsation of small intracranial aneurysms.

**Variables**	**Univariable**		**Multivariable**	
	**OR (95% CI)**	***p*-value**	**OR (95% CI)**	***p*-value**
Aneurysm size	1.67 (1.32–2.13)	<0.001		
Neck size	1.36 (1.00–1.84)	0.048		
Perpendicular height	1.67 (1.29–2.15)	<0.001		
Aneurysm height	1.8 (1.40–2.32)	<0.001		
Vessel size	0.66 (0.48–0.90)	0.008		
Size ratio	4.66 (2.51–8.63)	<0.001	2.59 (1.34–4.99)	0.005
Aspect ratio	4.18 (1.83–9.58)	0.001		
Flow angle	1.01 (1.00–1.02)	0.029		
**Aneurysm location**				
MCA vs. ICA	3.66 (1.40–9.52)	0.008		
ACA/PCoA/PC vs. ICA	2.03 (1.11–3.71)	0.022		
Irregular aneurysm	10.92 (4.85–24.60)	<0.001	6.44 (2.69–15.40)	<0.001
Aneurysm rupture	12.74 (2.77–58.58)	0.001	7.05 (1.33–37.24)	0.022

*MCA, middle cerebral artery; ACA, anterior cerebral arteries (including anterior cerebral artery and anterior communicating artery); PCoA, posterior communicating artery; PC, posterior circulation*.

## Discussion

This was a prospective study on small intracranial aneurysms detected by 4D-CTA. We found that irregular pulsation, non-carotid artery aneurysm, and larger flow angle were independently associated with small aneurysm rupture. Meanwhile, aneurysm rupture, irregular aneurysm, and larger size ratio were independently associated with presence of irregular pulsation. Our findings suggest that the irregular pulsation detected by 4D-CTA could be used to potentially evaluate the risk of small aneurysm rupture in a clinical setting.

4D-CTA provides dynamic information on aneurysm wall and visualizes wall motion during cardiac cycle. Using 4D-CTA images, we found that ruptured small aneurysms more often showed irregular pulsation, and that irregular pulsation was independently associated with aneurysm rupture. This finding is similar those of some studies that reported irregular pulsation was correlated with the status of aneurysm rupture (10, 17). Our study focused on prediction of small aneurysm rupture using a large sample size in a prospective design. Aneurysms with irregular pulsation had varied thickness and absence of the internal elastic lamina and tunica media (18). The irregular pulsation was reported to be associated with a morphologic change in shape at follow-up for identifying aneurysms with a high rupture risk (19). Krings et al. (20) reported that the presence of irregular pulsation was associated with aneurysm growth. Recently, Ferrari et al. found that 5 of 9 aneurysms with irregular pulsation had a significant correlation with dark-reddish thinner wall (21). The point of irregular pulsation of the aneurysm wall may indicate the potential rupture point (18). Therefore, the irregular pulsation of aneurysms may suggest that these aneurysms are unstable, indicating that irregular pulsation more potentially leads to aneurysm growth and rupture of small aneurysms.

Different from our previous study on associations of irregular pulsation of asymptomatic UIAs with conventional risk factors (14), this study showed that irregular pulsation more often occurred in a ruptured aneurysm. The site of a ruptured aneurysm may correspond to the area of irregular pulsation (13, 17). We also found that conventional rupture-related characteristics, such as aneurysm irregularity and size ratio, were independent predictors of irregular pulsation. Larger size ratio increases areas of low aneurysmal wall shear stress and causes more complex flow patterns within an aneurysm, which may lead to aneurysm rupture (15). Aneurysm irregularity also reflects the focal weakness of the wall of the aneurysm, which is related to degeneration. These changes may lead to a variety of aneurysm wall remodeling, including smooth muscle cell migration, endothelial damage, and inflammatory cell infiltration. Irregular pulsation will more likely develop, but underlying mechanisms require further studies.

Moreover, we found that non-internal carotid artery aneurysms were independently associated with small aneurysm rupture. This finding is similar to previous studies that have shown that aneurysm location is a risk factor of aneurysm rupture. The International Study of Unruptured Intracranial Aneurysms (ISUIA) has shown that the rupture rate of aneurysms varies depending on aneurysm location (1). Aneurysms located in the anterior cerebral artery, posterior communicating artery, and posterior circulation have a relatively high risk of rupture regardless of small size (4, 13, 22). The flow angle indicating the spatial relationship between aneurysm dome and parent vessel may increase the risk of rupture by influencing intra-aneurysmal hemodynamics (23).

This study has several limitations. First, this was a single-center study on intracranial aneurysms examined by 4D-CTA. Although all the consecutive patients were prospectively collected, selection bias exists. Most patients with unruptured intracranial aneurysms are treated in our tertiary center, and the sample of ruptured aneurysms was relatively small. Aneurysms whose 4D-CTA images were of poor quality and not able to identify aneurysm pulsation were excluded. Second, pulsating changes may be a waving movement caused by motions or other artifacts (24). Measurement of aneurysm pulsation and volume changes in the aneurysms were not obtained in our workstation. Focal protuberance was independently identified using three consecutive frames by two neuroradiologists. Third, morphological characteristics and aneurysm pulsation may change before and after rupture. Our findings may represent the status of aneurysm rupture. Prospective longitudinal studies focusing on the relationship between irregular pulsation of aneurysm and aneurysm growth and rupture are needed. Nevertheless, we investigated the association of irregular pulsation with aneurysm rupture and conventional risk factors using prospective data.

Clinical characteristics and aneurysm characteristics were prospectively collected to determine the predictors of small aneurysm rupture. The irregular pulsation of aneurysms detected by 4D-CTA was associated with small aneurysm rupture. Ruptured aneurysm, irregular aneurysm, and larger size ratio were independently associated with irregular pulsation. The irregular pulsation of aneurysms may help evaluate the risk of rupture of small intracranial aneurysms.

## Data Availability Statement

The original contributions presented in the study are included in the article/supplementary material, further inquiries can be directed to the corresponding authors.

## Ethics Statement

The studies involving human participants were reviewed and approved by the Institutional Review Board of Renji Hospital, School of Medicine Shanghai Jiao Tong University. The patients/participants provided their written informed consent to participate in this study.

## Author Contributions

JZ, YC, and BL were involved in data collection and morphology measurement. JZ and QG drafted the manuscript. HZ, SD, and BZ were involved in data implementation. SD, YP, and JW critically corrected the manuscript. BZ and JW had the idea for study design. All authors read and approved the final version of the manuscript.

## Funding

This study was supported by the Shanghai Science and Technology Project (21Y11906200), National Facility for Translational Medicine (Shanghai, TMSK-2021-147), National Key Research and Development Program (2016YFC1300800), and SJTU Medical Engineering Cross-cutting Research Foundation (ZH2018ZDA07).

## Conflict of Interest

The authors declare that the research was conducted in the absence of any commercial or financial relationships that could be construed as a potential conflict of interest.

## Publisher's Note

All claims expressed in this article are solely those of the authors and do not necessarily represent those of their affiliated organizations, or those of the publisher, the editors and the reviewers. Any product that may be evaluated in this article, or claim that may be made by its manufacturer, is not guaranteed or endorsed by the publisher.

## References

[1] Wiebers DO Whisnant JP Huston J III Meissner I Brown RD Jr. Unruptured intracranial aneurysms: natural history, clinical outcome, and risks of surgical and endovascular treatment. Lancet. (2003) 362:103–10. 10.1016/S0140-6736(03)13860-3 12867109

[2] Sonobe M Yamazaki T Yonekura M Kikuchi H. Small unruptured intracranial aneurysm verification study: SUAVe study, Japan. Stroke. (2010) 41:1969–77. 10.1161/STROKEAHA.110.585059 20671254

[3] Greving JP Wermer MJ Brown RD Jr Morita A Juvela S. Development of the PHASES score for prediction of risk of rupture of intracranial aneurysms: a pooled analysis of six prospective cohort studies. Lancet Neurol. (2014) 13:59–66. 10.1016/S1474-4422(13)70263-1 24290159

[4] Ikawa F Morita A Tominari S Nakayama T Shiokawa Y Date I. Japan Neurosurgical Society for UCAS Japan Investigators Rupture risk of small unruptured cerebral aneurysms. J Neurosurg. (2020) 132:69–78. 10.3171/2018.9.JNS181736 30684948

[5] Etminan N Beseoglu K Steiger HJ Hanggi D. The impact of hypertension and nicotine on the size of ruptured intracranial aneurysms. J Neurol Neurosurg Psychiatry. (2011) 82:4–7. 10.1136/jnnp.2009.199661 20667856

[6] Xu T Lin B Liu S Shao X Xia N Zhang Y. Larger size ratio associated with the rupture of very small (< /=3 mm) anterior communicating artery aneurysms. J Neurointerv Surg. (2017) 9:278–82. 10.1136/neurintsurg-2016-012294 27009240

[7] Malhotra A Wu X Geng B Hersey D Gandhi D Sanelli P. Management of small unruptured intracranial aneurysms: a survey of neuroradiologists. AJNR. (2018) 39:875–80. 10.3174/ajnr.A5631 29650787 PMC7410652

[8] Varble N Tutino VM Yu J Sonig A Siddiqui AH Davies JM. Shared and distinct rupture discriminants of small and large intracranial aneurysms. Stroke. (2018) 49:856–64. 10.1161/STROKEAHA.117.019929 29535267 PMC5871584

[9] Dissaux B Ognard J Cheddad El Aouni M Nonent M Haioun K Magro E. Volume variation may be a relevant metric in the study of aneurysm pulsatility: a study using ECG-gated 4D-CTA (PULSAN). J Neurointerv Surg. (2020) 12:632–6. 10.1136/neurintsurg-2019-015336 31699886

[10] Gu Y Zhang Y Luo M Zhang H Liu X Miao C. Risk factors for asymptomatic intracranial small aneurysm rupture determined by electrocardiographic-gated 4D computed tomographic (CT) angiography. Med Sci Monit. (2020) 26:e921835. 10.12659/MSM.921835 31942867 PMC6984014

[11] Bouillot P Brina O Chnafa C Cancelliere NM Vargas MI Radovanovic I. Robust cerebrovascular blood velocity and flow rate estimation from 4D-CTA. Med Phys. (2019) 46:2126–36. 10.1002/mp.13454 30793326

[12] Vanrossomme AE Eker OF Thiran JP Courbebaisse GP Boudjeltia KZ. Intracranial aneurysms: wall motion analysis for prediction of rupture. AJNR. (2015) 36:1796–802. 10.3174/ajnr.A4310 25929878 PMC7965030

[13] Hayakawa M Katada K Anno H Imizu S Hayashi J Irie K. CT angiography with electrocardiographically gated reconstruction for visualizing pulsation of intracranial aneurysms: identification of aneurysmal protuberance presumably associated with wall thinning. AJNR Am J Neuroradiol. (2005) 26:1366–9. 15956499 PMC8149081

[14] Zhang J Li X Zhao B Zhang J Sun B Wang L. Irregular pulsation of intracranial unruptured aneurysm detected by four-dimensional CT angiography is associated with increased estimated rupture risk and conventional risk factors. J Neurointerv Surg. (2021) 13:854–9. 10.1136/neurintsurg-2020-016811 33472873

[15] Chen Y Lin B Zhou J Chen L Yang Y Zhao B. Morphological predictors of middle cerebral artery bifurcation aneurysm rupture. Clin Neurol Neurosurg. (2020) 192:105708. 10.1016/j.clineuro.2020.105708 32058208

[16] Chen Y Xing H Lin B Zhou J Ding S Wan J. Morphological risk model assessing anterior communicating artery aneurysm rupture: development and validation. Clin Neurol Neurosurg. (2020) 197:106158. 10.1016/j.clineuro.2020.106158 32836062

[17] Hayakawa M Maeda S Sadato A Tanaka T Kaito T Hattori N. Detection of pulsation in ruptured and unruptured cerebral aneurysms by electrocardiographically gated 3-dimensional computed tomographic angiography with a 320-row area detector computed tomography and evaluation of its clinical usefulness. Neurosurgery. (2011) 69:843–51; discussion 851. 10.1227/NEU.0b013e318225b2d3 21623246

[18] Kato Y Hayakawa M Sano H Sunil MV Imizu S Yoneda M. Prediction of impending rupture in aneurysms using 4D-CTA: histopathological verification of a real-time minimally invasive tool in unruptured aneurysms. Minim Invasive Neurosurg. (2004) 47:131–5. 10.1055/s-2004-818492 15343426

[19] Hayakawa M Tanaka T Sadato A Adachi K Ito K Hattori N. Detection of pulsation in unruptured cerebral aneurysms by ECG-gated 3D-CT angiography (4D-CTA) with 320-row area detector CT (ADCT) and follow-up evaluation results: assessment based on heart rate at the time of scanning. Clin Neuroradiol 24. (2014) 145-50. 10.1007/s00062-013-0236-8 23913018

[20] Krings T Willems P Barfett J Ellis M Hinojosa N Blobel J. Pulsatility of an intracavernous aneurysm demonstrated by dynamic 320-detector row CTA at high temporal resolution. Central Eur Neurosurg. (2009) 70:214–8. 10.1055/s-0029-1225355 19851955

[21] Ferrari F Cirillo L Calbucci F Bartiromo F Ambrosetto P Fioravanti A. Wall motion at 4D-CT angiography and surgical correlation in unruptured intracranial aneurysms: a pilot study. J Neurosurg Sci. (2019) 63:501–8. 10.23736/S0390-5616.16.03640-7 27188661

[22] Bijlenga P Ebeling C Jaegersberg M Summers P Rogers A Waterworth A. Risk of rupture of small anterior communicating artery aneurysms is similar to posterior circulation aneurysms. Stroke. (2013) 44:3018–26. 10.1161/STROKEAHA.113.001667 23899912

[23] Baharoglu MI Schirmer CM Hoit DA Gao BL Malek AM. Aneurysm inflow-angle as a discriminant for rupture in sidewall cerebral aneurysms: morphometric and computational fluid dynamic analysis. Stroke. (2010) 41:1423–30. 10.1161/STROKEAHA.109.570770 20508183

[24] Matsumoto M Sasaki T Suzuki K Sakuma J Endo Y Kodama N. Visualizing the dynamics of cerebral aneurysms with four-dimensional computed tomographic angiography. Neurosurgery. (2006) 58:E1003; author reply E1003. 10.1227/01.NEU.0000217323.32623.F7 16639292

